# Vitamin D and risk of developing type 2 diabetes in the SUN project: a prospective cohort study

**DOI:** 10.1007/s40618-024-02324-3

**Published:** 2024-03-08

**Authors:** A. Valer-Martinez, C. Sayon-Orea, J. A. Martinez, F. J. Basterra-Gortari, M. A. Martinez-Gonzalez, M. Bes-Rastrollo

**Affiliations:** 1 Department of Preventive Medicine and Public Health, University of Navarra, Pamplona, Spain; 2 Department of Family Medicine, Aragon Health Service (SALUD), Zaragoza, Spain; 3 CIBERobn, Instituto de Salud Carlos III, Madrid, Spain; 4 IdiSNa, Navarra Institute for Health Research, Universidad de Navarra, C/Irunlarrea, 1 (Ed. Investigación), 31008 Pamplona, Navarra Spain; 5 Navarra Public Health Institute, Pamplona, Spain; 6 Institute IMDEA Food, Madrid, Spain; 7 Department of Nutrition, Food Science and Physiology/Centre for Nutrition Research, University of Navarra, Pamplona, Spain; 8 Department of Endocrinology and Nutrition, Hospital Universitario de Navarra, Universidad Pública de Navarra, Pamplona, Spain; 9 Harvard T.H. Chan School of Public Health, Boston, MA USA

**Keywords:** Predicted vitamin D, Type 2 diabetes, SUN project, Prospective cohort

## Abstract

**Purpose:**

Vitamin D deficiency has been associated with multiple chronic diseases, including metabolic disorders such as insulin resistance and type 2 diabetes (T2D). The aim of the study was to analyze the association between validated predicted serum vitamin D status and the risk of developing T2D in a large prospective cohort based on a Mediterranean population.

**Methods:**

The SUN project is a prospective and dynamic Spanish cohort that gathers university graduates who have answered lifestyle questionnaires, including a validated Food Frequency Questionnaire. The association between predicted serum vitamin D and the risk of T2D was assessed through Cox regression models according to quartiles (Q) of predicted vitamin D at baseline. The models were adjusted for potential confounders and sensitivity analyses were performed to ensure the robustness of our findings.

**Results:**

Our study included a total of 18,594 participants and after a total follow-up of 238,078 person-years (median follow-up of 13.5 years), 209 individuals were diagnosed with incident T2D. We found a significant inverse association between predicted levels of serum vitamin D and the risk of developing T2D, after adjusting for potential confounders and performing different sensitivity analyses (hazard ratio Q4 vs. Q1: 0.48, 95% CI 0.26–0.88; *p* for trend = 0.032).

**Conclusion:**

The outcomes suggest that higher levels of vitamin D at baseline may be associated with a reduced risk of developing T2D.

**Supplementary Information:**

The online version contains supplementary material available at 10.1007/s40618-024-02324-3.

## Introduction

Type 2 diabetes (T2D) is the most common type of diabetes and a highly prevalent chronic disease, which leads to an increased morbidity affecting life quality and functional capacities of many patients, along with higher rates of mortality [1]. The last report published in 2021 by the International Diabetes Federation (IDF) stated that approximately 537 million adults between 20 and 79 years old had already been diagnosed with diabetes worldwide and 541 million adults were at high risk of developing type 2 diabetes [1, 2]. In addition, such reports have predicted that cases of diabetes are expected to rise to 643 million by 2030 and 783 million by 2045 [2]. Furthermore, the continuous growing trend in the number of cases has generated a significant increase in health care costs, which has turned T2D into a relevant public health issue [2].

Vitamin D deficiency has also become a worldwide health problem. In fact, published scientific evidence highlights that near 40% of Europeans and 24% of North Americans show deficient values of serum vitamin D, although data may vary depending on age or ethnicity in different regions [3–6]. Vitamin D is a fat-soluble vitamin, which is naturally found in different dietary sources (such as fatty fish or egg yolks), and it is also synthesized endogenously after sun exposure [7, 8]. Vitamin D has been involved in many different physiological processes. Apart from its well-known role in calcium and bone metabolism, it seems to be associated with the regulation of inflammatory processes as well as the modulation of different mechanisms such as cell growth, immune function, and glucose metabolism [9, 10]. In fact, there is current evidence that vitamin D deficiency plays a role in the development of metabolic disorders including insulin resistance and T2D [11]. Several observational studies have shown a possible association between vitamin D deficiency and diabetes onset as well as progression toward complications [12–14]. In fact, various mechanisms have been described such as the modulation of immune responses and depletion of systemic inflammation, the reduction of peripheral insulin resistance through vitamin D receptors located in muscles and liver or the increase or calcium influx into pancreatic beta cells, which influences insulin secretion, among others [15, 16]. However, it remains unclear the protective role of vitamin D supplementation in the prevention of T2D [17–21]. Two recent trials showed that the risk of a new onset of T2DM was lower in the supplemented group, although the differences were not statistically significant [17, 18]. The most important randomized trial (the D2d study) observed a 12% relative reduction in the risk of T2D [17], whereas The Diabetes Prevention with Active Vitamin D study found a 13% lower risk of T2D among adults with prediabetes after supplementation with eldecalcitol [18].

This study aims to give some light over the possible association between levels of predicted serum vitamin D and the risk of developing type 2 diabetes in a large Mediterranean cohort with a long follow-up period.

## Materials and methods

### Study population

The SUN project (Seguimiento Universidad de Navarra) is a prospective, multipurpose, and dynamic cohort conducted in Spain that started to recruit participants in 1999 [22].

**Fig. 1:**
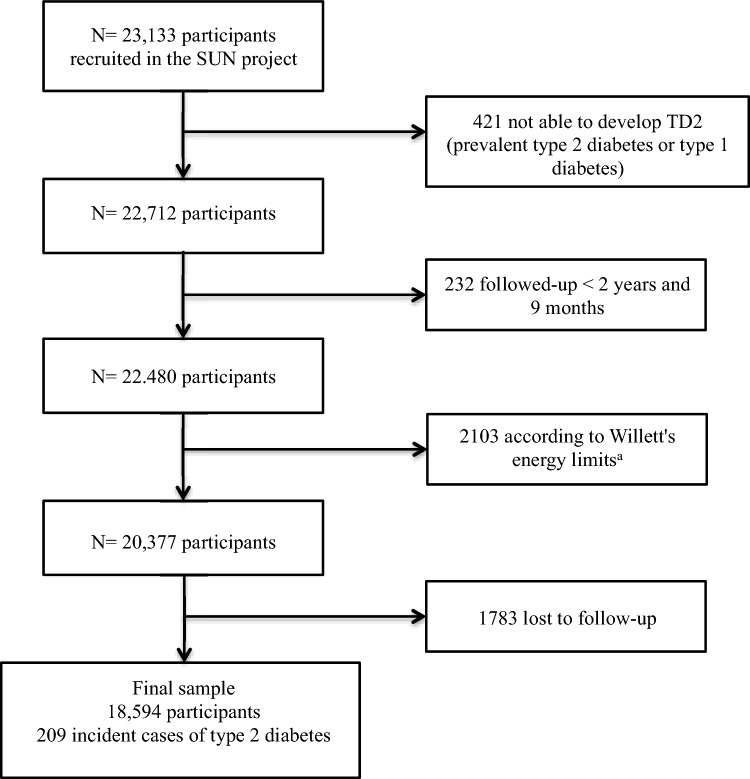
Flowchart of participants included in the present analyses. ^a^Willett’s energy limits (< 800 kcal/d or > 4000 kcal/d in men and < 500 kcal/d or > 3500 kcal/d in women)

Information was collected from participants through validated questionnaires, which were mailed at baseline and every 2 years. They included information about socio-demographic, lifestyle and dietary variables, as well as the prevalence or incidence of different diseases at baseline and during the follow-up. All participants are university graduates and over the half are health professionals who live throughout Spain, which provides a wide range of different lifestyles and dietary patterns. Up to May 31, 2022, a total of 23,133 participants completed their baseline questionnaire. For the current analysis, the sample was selected according to the following exclusion criteria: 421 individuals with a previous diagnosis of diabetes at the time of the enrollment were excluded; we also excluded 232 participants who completed the baseline questionnaire after August 31, 2019 to ensure a minimum follow-up of 2 years and 9 months. We further excluded 2103 participants with energy intake outside the predefined limits by Willett (a daily energy intake below 500 kcal or above 3500 kcal for women and below 800 kcal or above 4000 kcal for men) [23] and also 1783 subjects who were lost to follow-up. Finally, a total of 18,594 participants and 209 incident cases of type 2 diabetes were included (Fig. 1). The overall long-term retention rate in the cohort was 91%. The institutional review committee of the University of Navarra approved the study. Voluntary completion of the first questionnaire was considered to imply informed consent.

### Exposure assessment

Predicted vitamin D serum levels were forecasted through a previously validated predictive model obtained after multiple linear regression analysis [24]. The prediction model included the following variables: dietary intake of vitamin D; age and sex; body mass index (BMI), which was calculated from self-reported weight and height (kg/m^2^); skin reaction after sun exposure (mild or severe reaction); time spent on daily walks (minutes per day); and summer sun exposure (hours per day) (Supplementary Table 1). This last variable was estimated after performing a weighted average of the weekly sun exposure during summer. Dietary information was obtained from a validated semiquantitative 136-item Food Frequency Questionnaire (FFQ) [25–27]. In this way, values of vitamin D ingested from diet and vitamin supplements were included in the same variable and it was adjusted for total energy intake (kcal) using the residual method. Physical activity was also assessed through a validated questionnaire [28]. Each practice of physical activity was weighted using its proportional number of metabolic equivalents (METs) and time spent on each activity was also taken into account to obtain a global value of METs-h/week for each volunteer. The whole process of the abovementioned predictive model has been previously described [24].

### Outcome assessment

Ascertainment of type 2 diabetes mellitus (T2D) in the SUN cohort has been well described before [29]. Prevalent cases of T2D were initially classified only as probable cases when the participant reported a confirmed medical diagnosis at baseline. Adjudication of incident cases of T2D were all those participants who were medically diagnosed of diabetes in one of the follow-up questionnaires but did not report the disease at baseline. In addition, these volunteers were asked to fill in an additional confirmation questionnaire and their medical records were also requested. Subsequently, an independent expert endocrinologist who was blinded to the exposure definitively confirmed the incident cases of T2D, based on all this information collected. The incident cases of T2D were adjudicated following all these steps and using the American Diabetes Association’s criteria [30]. These criteria include: fasting plasma glucose ≥ 126 mg/dL (7.0 mmol/L) or 2-h plasma glucose ≥ 200 mg/dL (11.1 mmol/L) during oral glucose tolerance test or glycated hemoglobin ≥ 6.5% or a random plasma glucose ≥ 200 mg/dL (11.1 mmol/L) in a patient with classic symptoms of hyperglycemia or hyperglycemic crisis [30]**.**

### Covariates assessment

The baseline questionnaire gathered information about socio-demographics (marital status, educational level), validated anthropometric measures [31], lifestyle habits (smoking status, physical activity or time spent watching television), and medical variables (prevalence of cancer, hypertension, hypertriglyceridemia and hypercholesterolemia, among others). Information about the total amount of energy intake (kcal/day) was also collected. Adherence to Mediterranean diet (MedDiet) was evaluated using the well-known score proposed by Trichopoulou et al. [32]. This score ranged from 0 to 9 points; where higher score implies higher adherence to the Mediterranean diet.

### Statistical analysis

Description of baseline characteristics was performed, computing means and standard deviations for continuous variables and proportions for categorical variables, and comparison between quartiles of predicted serum vitamin D was conducted using ANOVA test for continuous variables and Chi-squared test for categorical variables. Afterward, *p* values were adjusted performing Bonferroni’s method. The association between quartiles of predicted serum vitamin D levels and the risk of developing T2D was assessed using Cox regression models, with the lowest quartile as the reference category. Hazard ratios (HR) and their 95% confidence intervals (95% CI) were estimated. Moreover, we calculated the continuous association of incident T2D for each 10 ng/mL increase of predicted serum levels of vitamin D.

A first model was adjusted for age and sex. A second model, included additionally, marital status (married/others), smoking status (current, former or never smoker), cumulative exposure to tobacco (smoking pack-years), weight change (yes/no), years of university education attained, TV hours/day, family history of T2D (yes/no), physical activity (MET-h/week), adherence to Mediterranean dietary pattern (low (0–3), moderate (4–6), high (7–9)), energy intake (kcal/day), sugar-sweetened beverage consumption (servings/day), snacking between meals (yes/no), following a special diet (yes/no), prevalent hypertension, prevalent cancer, prevalent hypercholesterolemia, prevalent hypertriglyceridemia. A third model was fitted additionally adjusting for obesity (≥ 30 kg/m^2^ yes/no). Missing values were imputed (simple imputation) using the Stata command *impute*, based on multivariable linear regression models for continuous variables and multivariable logistic or multinomial regression models for categorical variables. The imputed variables were time spent watching TV (18.0% of missing values), following a special diet (2.4%), skin reaction after sun exposure (1.6%), and summer sun exposure (20.7%). We performed an analysis to assess potential effect modification by sex, age (under or above 50 years old), and obesity (yes/no). We used the likelihood ratio test comparing the fully adjusted Cox regression model and the same model with the interaction cross product terms with quartiles of estimated vitamin D (3 d.f,) to estimate the p value for interaction. Several sensitivity analyses were conducted under different assumptions: excluding extreme values of total energy intake (< *p*1 and > *p*99), excluding participants with outliers’ values of predicted serum vitamin D (± 1.5 IQR). Additional analyses were performed excluding those participants who had prevalent cancer, hypertension, hypertriglyceridemia, and hypercholesterolemia to reduce the chance of reverse causality. We also conducted analyses including only participants with family history of T2D, overweight (BMI ≥ 25 kg/m^2^) or sedentary lifestyle, which was defined as physical activity below the median (MET-h/week < *p*50).

Restricted cubic splines with three knots, considering 10 (ng/mL) as the reference, were applied to the flexible model to graphically represent the dose–response association between type 2 diabetes and the predicted serum vitamin D level (as a continuous variables), as well as to evaluate non-linearity. All *p* values were two-tailed and statistical significance was set at the cut-off point of *p* < 0.05. All analyses were performed using STATA 15.0 (StataCorp, College Station, TX).

## Results

**Fig. 2:**
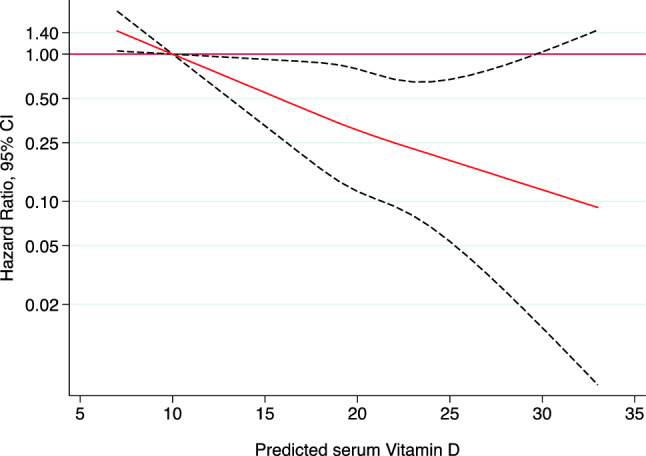
Restricted cubic splines dose–response pattern: adjusted hazard ratios (HR)* and 95% confidence intervals (CI) for the development of T2D according to predicted serum vitamin D. *Adjusted for sex, age, marital status, smoking status (current, former or never smoker), smoking pack-years, weight change, years of university, TV hours/day, family history of T2D, physical activity (MET-h/week), Trichopoulou’s 9-point score/Mediterranean dietary pattern, energy intake (kcal/day), sugar-sweetened beverage consumption (servings/day), snacking, following a special diet, prevalent hypertension, prevalent cancer, prevalent hypercholesterolemia, prevalent hypertriglyceridemia and obesity (>= 30 kg/m^2^ yes, no)

For the analysis, we included 18,594 participants, with a 60.5% of female participation; the mean age was 38.0 ± 12.2 years. Table 1 describes baseline characteristics of the sample according to quartiles of predicted serum vitamin D. Mean predicted serum vitamin D was 19.9 ± 2.32 ng/mL. Participants in the highest quartile of predicted vitamin D tended to be younger, mostly not married and they showed lower prevalence of cardiovascular risk factors as well as cancer. They also tended to smoke less, to show lower body mass index, to be more physically active (3.7 times more sportive as compared to those in the first quartile), and to have greater summer sunlight exposure. In terms of nutrition, they also showed greater daily intake of vitamin D and slightly higher adherence to Mediterranean diet compared to the first quartile. Other characteristics such as time spent watching TV or sleeping, as well as the intake of the different nutrients (carbohydrates, proteins and fat) and alcohol were very similar across quartiles. After a total of 238,078 person-years with a median follow-up of 13.5 years, 209 individuals were diagnosed with incident type 2 diabetes. Most of the T2D cases (*n* = 118) were found in the first quartile of predicted serum vitamin D, showing an incident rate of 2 × 10^–3^ vs. 0.3 × 10^–3^ in the last quartile. When comparing quartiles of predicted serum vitamin D, considering the lowest quartile as the reference, we found an inverse significant association between predicted levels of serum vitamin D and the risk of developing diabetes, after adjusting for potential cofounders (Table 2). Compared to the reference category, the hazard ratios shown were HR_Q2vsQ1_ of 0.65 (95% CI 0.44–0.95), HR_Q3vsQ1_ of 0.71 (95% CI 0.45–1.10), HR_Q4vsQ1_ of 0.48 (95% CI 0.26–0.88) *p* for trend = 0.032. In addition, each increase of 10 ng/mL (25 nmol/L) in 25(OH)D concentration was associated with a HR of 0.31 (95% CI 0.14–0.68) Fig. 2 represents the dose–response relationship between the predicted serum levels of vitamin D and the risk of developing T2D. The graph shows that serum vitamin D levels under 10 ng/mL (25 nmol/L) seemed to be significantly associated with an increased risk of developing T2D up to 40%. However, as serum vitamin D increases, the curve suggested a statistically significant protective effect.

**Table 1:** Baseline characteristics according to quartiles of predicted serum vitamin D

	Quartiles of predicted serum vitamin D				*p* value*
	Q1	Q2	Q3	Q4	
*N*	4649	4648	4649	4648	
Serum vitamin D predicted status (range, ng/mL)	7.1;18.6	18.7;19.8	19.9;21.1	21.2;32.7	
Age (years) (SD)	41.9 (12.1)	39.2 (11.6)	36.4 (11.7)	34.7 (12.1)	< 0.001
Women (%)	62.1	60.8	60.4	58.9	0.544
Smoking status (%)					< 0.001
Never	43.9	46.4	50.9	55.0	
Current	21.0	22.8	22.3	21.4	
Former	35.1	30.9	26.9	23.7	
Marital status, married (%)	61.1	55.3	45.6	38.1	< 0.001
Years of university (years) (SD)	5.2 (1.6)	5.2 (1.5)	5.1 (1.5)	4.9 (1.4)	< 0.001
Body mass index (kg/m^2^) (SD)	26.1 (3.9)	23.5 (2.8)	22.5 (2.8)	21.9 (2.7)	< 0.001
Weight change (%)^a^	44.0	31.8	23.9	21.4	< 0.001
Physical activity (METs-h/wk) (SD)	10.7 (11.8)	15.1 (13.3)	21.8 (16.4)	39.7 (32.0)	< 0.001
TV (hours/day) (SD)	1.6 (1.1)	1.6 (1.1)	1.6 (1.1)	1.7 (1.4)	< 0.001
Siesta (hours/day) (SD)	0.6 (0.5)	0.6 (0.5)	0.5 (0.5)	0.5 (0.5)	< 0.001
Sleeping hours (hours/day) (SD)	7.2 (0.9)	7.3 (0.8)	7.3 (0.8)	7.3 (0.9)	< 0.001
Walking time (min/day) (SD)	22.5 (18.4)	25.5 (18.6)	35.7 (23.5)	65.7 (40.5)	< 0.001
Summer sun exposure (h/day) (SD)	0.6 (0.6)	0.8 (0.7)	1.2 (0.9)	2.1 (1.8)	< 0.001
Skin reaction after sun exposure (%)					< 0.001
Mild reaction	74.8	98.3	99.1	99.3	
Severe reaction	25.2	1.7	0.9	0.7	
Energy intake (kcal/d) (SD)	2282 (620)	2293 (601)	2359 (601)	2429 (625)	< 0.001
Carbohydrate intake (% of energy)	43.0 (7.8)	43.3 (7.4)	43.8 (7.3)	44.0 (7.6)	< 0.001
Protein intake (% of energy/d)	18.5 (3.5)	18.2 (3.3)	18.2 (3.2)	18.2 (3.2)	< 0.001
Fat intake (% of energy)	36.4 (6.8)	36.4 (6.5)	36.1 (6.4)	36.0 (6.7)	0.068
Monounsaturated fatty acids intake (% of energy)	16.0 (3.9)	15.9 (3.7)	15.7 (3.6)	15.5 (3.7)	< 0.001
Saturated fatty acids intake (% of energy)	12.5 (3.2)	12.6 (3.1)	12.5 (3.2)	12.3 (3.3)	< 0.001
Polyunsaturated fatty acids intake (% of energy)	5.2 (1.6)	5.2 (1.6)	5.2 (1.5)	5.2 (1.6)	0.999
Trichopoulou’s 9-point score/Mediterranean dietary pattern (SD)	4.2 (1.8)	4.1 (1.8)	4.2 (1.8)	4.4 (1.8)	< 0.001
Following special diet (%)	10.5	7.0	6.2	7.5	< 0.001
Between-meal snacking (%)	36.7	30.9	31.7	33.4	< 0.001
Alcohol intake (g/d) (SD)	6.9 (11.2)	6.7 (10.1)	6.5 (9.3)	6.4 (9.3)	0.999
Sugar-sweetened beverage (servings/day)	0.2 (0.4)	0.2 (0.4)	0.2 (0.4)	0.2 (0.4)	0.68
Total vitamin D intake (mcg/d)^b^	6.1 (4.1)	6.3 (4.4)	6.9 (5.2)	7.7 (7.5)	< 0.001
Total dietary fiber intake (g/d)	22.0 (10.1)	21.6 (9.3)	22.7 (9.6)	23.9 (10.4)	< 0.001
Family history of T2D (%)	19.4	15.6	13.3	13.2	< 0.001
Prevalent hypertension (%)	16.3	10.3	8.5	7.1	< 0.001
Prevalent CVD (%)	1.5	1.3	1.4	1.5	0.999
Prevalent cancer (%)	3.4	2.8	2.4	1.9	< 0.001
Prevalent hypercholesterolemia (%)	21.7	18.0	14.5	13.5	< 0.001
Prevalent hypertriglyceridemia (%)	9.8	6.8	4.8	4.4	< 0.001

Continuous variables are described as means (standard deviation) and categorical variables as percentages

*p values adjusted with Bonferroni’s method

^a^Weight gain prior baseline of 5 or more kgs

^b^Total intake of dietary vitamin D and supplementation, energy adjusted by residual method (mcg/d)

**Table 2:** Cox proportional HRs and 95% CI for incident T2D according to quartiles of baseline predicted serum vitamin D

	Q1	Q2	Q3	Q4	*p* trend	Each 10 ng/mL^d^
Predicted vit D^a^	17.62 (16.51;18.23)	19.27 (18.96;19.56)	20.44 (20.12;20.80)	22.39 (21.69;23.48)		
Cases of incident T2D	118	43	32	16		
Person-years	58,110	60,500	60,422	59,046		
Incident rate 10^–3^	2.0	0.7	0.5	0.3		
Age- and sex-adjusted model	1.00 (reference)	0.40 (0.28–0-57)	0.35 (0.24–0.52)	0.20 (0.12–0.34)	< 0.001	0.07 (0.04–0.13)
Multiple-adjusted model1^b^	1.00 (reference)	0.46 (0.32–0.67)	0.48 (0.31–0.74)	0.32 (0.17–0.57)	< 0.001	0.11 (0.06–0.23)
Multiple-adjusted model2^c^	1.00 (reference)	0.65 (0.44–0.95)	0.71 (0.45–1.10)	0.48 (0.26–0.88)	0.032	0.31 (0.14–0.68)

^a^Predicted vitamin D status (ng/mL) expressed by *p*50 (*p*25;*p*75)

^b^Model adjusted for sex, age, marital status, smoking status (current, former or never smoker), smoking pack-years, weight change, years of university, TV hours/day, family history of T2D, physical activity (MET-h/week), Trichopoulou’s 9-point score/Mediterranean dietary pattern, energy intake (kcal/day), sugar-sweetened beverage consumption (servings/day), snacking, following a special diet, prevalent hypertension, prevalent cancer, prevalent hypercholesterolemia, prevalent hypertriglyceridemia

^c^Model 1 additionally adjusted for obesity (>=30 kg/m^2^ yes, no)

^d^HR and 95% CI for incident T2D for each 10 ng/mL increase of predicted vitamin D

We carried out several sensitivity analyses to determine the robustness of our findings. After excluding patients with prevalent cancer, hypertension, hypertriglyceridemia, extreme daily energy intake and the outliers of predicted vitamin D and including only participants with sedentary lifestyle and overweight, the results did not suffer any substantial change compared to the main analyses (see Table 3). The inverse association between predicted 25(OH)D (highest vs. lowest quartile) and the risk of incident T2D was stronger after excluding participants diagnosed with hypertension at baseline (HR_Q4vsQ1_ 0.28; 95% CI 0.11–0.72) and including only individuals with sedentary habits (HR_Q4vsQ1_ 0.28; 95% CI 0.09–0.93) and overweight (HR_Q4vsQ1_ 0.19; 95% CI 0.07–0.56). However, when we excluded participants with prevalent hypercholesterolemia and took into account only individuals with family history of T2D, we did not find any statistically significant association (HR_Q4vsQ1_ 0.50; 95% CI 0.22–1.17 and HR_Q4vsQ1_ 0.34; 95% CI 0.10–1.10, respectively).

**Table 3:** Sensitivity analyses: HR and 95% CI (Q4 vs. Q1) for incident type 2 diabetes according to baseline predicted serum vitamin D

	*N*	Incident T2D	HR (95% CI)
Main analysis Q4 vs. Q1^a^	18,594	209	0.48 (0.26–0.88)
Excluding participants with cancer at baseline^b^	18,103	198	0.48 (0.26–0.89)
Excluding participants with hypertension at baseline^c^	16,631	113	0.28 (0.11–0.72)
Excluding participants with hypertriglyceridemia at baseline^d^	17,393	136	0.44 (0.20–0.99)
Excluding participants with hypercholesterolemia at baseline^e^	15,446	114	0.50 (0.22–1.17)
Excluding extreme daily energy intake (< *p*1 or > *p*99)	20,108	217	0.50 (0.27–0.90)
Excluding outliers^f^	17,823	192	0.45 (0.24–0.86)
Including only participants with family history of T2D	2854	80	0.34 (0.10–1.10)
Including only overweight participants^g^	5538	173	0.19 (0.07–0.56)
Including only participants with sedentary lifestyle^h^	9297	135	0.28 (0.09–0.93)

^a^Adjusted for sex, age, marital status, smoking status (current, former or never smoker), smoking pack-years, weight change, years of university, TV hours/day, family history of T2D, physical activity (MET-h/week), Trichopoulou’s 9-point score/Mediterranean dietary pattern, energy intake (kcal/day), sugar-sweetened beverage consumption (servings/day), snacking, following a special diet, prevalent hypertension, prevalent cancer, prevalent hypercholesterolemia, prevalent hypertriglyceridemia, obesity (kg/m^2^)

^b^Without prevalent cancer adjustment

^c^Without prevalent hypertension adjustment

^d^Without prevalent hypertriglyceridemia adjustment

^e^Without prevalent hypercholesterolemia adjustment

^f^Without outliers located within ± 1.5 interquartile range of the average of predicted vitamin D

^g^Participants with BMI ≥ 25 kg/m^2^ were included

^h^Participants with sedentary lifestyle (MET-h/week < *p*50)

The stratified analysis for the effect modification (Table 4) showed a stronger association between predicted serum vitamin D levels and T2D in participants younger than 50 years, in men and in non-obese subjects when comparing the highest vs. the lowest quartile. However, we found no evidence of significant interaction between age (*p* for interaction 0.120), sex (*p* for interaction 0.961) or obesity (*p* for interaction 0.806).

**Table 4:** Analysis of effect modification: adjusted hazard ratios^a^ and 95% confidence intervals (CI) for the development of type 2 diabetes according to quartiles of predicted serum vitamin D in the SUN cohort stratified by potential confounders (age, sex, and obesity)

	N	Incident T2D	Q1	Q2	Q3	Q4	*p* for interaction
Age							0.120
Age ≥ 50 years	3292	127	1.00 (reference)	0.63 (0.38–1.03)	0.78 (0.44–1.36)	0.70 (0.34–1.43)	
Age < 50 years	15,302	82	1.00 (reference)	0.73 (0.40–1.34)	0.71 (0.34–1.50)	0.23 (0.06–0.85)	
Sex							0.961
Women	11,256	49	1.00 (reference)	0.78 (0.33–1.85)	0.96 (0.35–2.62)	0.54 (0.13–2.20)	
Men	7338	160	1.00 (reference)	0.57 (0.37–0.89)	0.63 (0.38–1.05)	0.45 (0.23–0.90)	
Obesity							0.806
Obese	844	73	1.00 (reference)	0.77 (0.31–1.93)	1.69 (0.59–4.85)	0.94 (0.11–8.22)	
Non-obese	17,750	136	1.00 (reference)	0.67 (0.43–1.03)	0.65 (0.39–1.08)	0.49 (0.25–0.95)	

^a^Adjusted for sex, age, marital status, smoking status (current, former or never smoker), smoking pack-years, weight change, years of university, TV hours/day, family history of T2D, physical activity (MET-h/week), Trichopoulou’s 9-point score/Mediterranean dietary pattern, energy intake (kcal/day), sugar-sweetened beverage consumption (servings/day), snacking, following a special diet, prevalent hypertension, prevalent cancer, prevalent hypercholesterolemia, prevalent hypertriglyceridemia, obesity (kg/m^2^)

## Discussion

In this prospective cohort of Spanish adults, the risk of developing T2D decreases in the highest quartiles of predicted serum vitamin D levels compared to the lowest quartile, independently of obesity and other potential confounders. After adjusting for obesity, the association was slightly attenuated remaining statistically significant, which may act as an intermediate factor in the risk of new-onset T2D. We did not adjust for BMI as a continuous variable because we assumed that it could be an intermediate link in the causal chain. Results showed a 52% reduction in the risk of developing T2D in the top vs. the bottom quartile of predicted vitamin D (HR 0.48; 95% CI 0.26–0.88, *p* = 0.032). Moreover, a relatively 69% lower incidence rate of type 2 diabetes after each increase of 10 ng/mL was observed. Our results are in agreement with previous large observational studies [12, 13]. Some meta-analyses based on observational studies mainly conducted in Nordic (Denmark, Norway and Sweden) or Anglo-Saxon countries have also reported an inverse association between directly measured serum 25(OH)D levels and the risk of incident diabetes [11–14, 33]. Afzal et al. conducted a meta-analysis of 16 studies and showed a 50% higher risk for incident diabetes in the lowest category compared to the highest one (OR 1.5; 95% CI 1.33–1.70) [12]. Another study also reported an increased risk in incident T2D of 22% after each decrease of 10 ng/mL (25 nmol/L) in serum 25(OH)D [34]. Song et al. included 21 studies and estimated a reduction of 4% in the risk of diabetes after each 10 nmol/L increment in serum 25(OH)D levels (*p* for linear trend = 0.0001), and when comparing the highest vs. the lowest category of 25(OH)D levels, they found a 38% risk reduction in T2D development (RR 0.62; 95% CI 0.54–0.70) [13]. However, the association between the two variables was also slightly decreased after adjustment for BMI [13]. In the same line, Rafiq et al. described an inverse correlation between levels of vitamin D and insulin resistance, stronger in the diabetic sample (*r* = −0.26; 95% CI −0.39 to −0.11, *p* = 0.001), independent of age and sex but enhanced after BMI’s increase [14]. These findings highlight obesity as part of the causal chain in the risk of new-onset T2D. In fact, the relationship between vitamin D and adiposity may be complex and bidirectional as obese patients tend to show an increased storage of 25(OH)D in adipose tissue and less sunlight exposure due to a reduced mobility, which contribute to low circulating 25(OH)D levels [13]. When we explored the non-linear association between predicted serum 25(OH)D and T2D, our results showed that values above 12 ng/mL (30 nmol/L) seemed to have a protective effect over the risk of developing T2D. This association remained statistically significant up to 30 ng/mL (75 nmol/L). It remains unclear what levels of 25(OH)D are necessary to influence glucose and insulin homeostasis, and therefore the risk of incident diabetes. Song et al. proposed that reaching levels of serum 25(OH)D of at least 50 nmol/L contributed to reduce the risk of T2D [13]. Moreover, Avila-Rubio et al. conducted their research in women with postmenopausal osteoporosis and found that levels of 25(OH)D above 45 ng/mL were necessary to balance glucose metabolism [35]. In this line, another research including 903 non-diabetic participants established the threshold of serum vitamin D at 30 ng/mL to reduce the incident rate of T2D [36]. There are different hypothesis that try to explain the mechanisms that lie beneath the association between vitamin-D-deficient status and a higher risk of diabetes. A large meta-analysis based on published small trials from 1980 to 2019 showed that sufficient levels of serum vitamin D diminish the risk of developing cellular pathological processes related to insulin resistance [15]. Such processes include the maintenance of low concentration of radicals, a low expression of pro-inflammatory cytokines but a higher production of anti-inflammatory ones [14, 15, 37]. Vitamin D is also involved in epigenetic processes affecting pancreatic β-cells and other insulin-sensitive peripheral tissues [15, 38]. Furthermore, vitamin has a modulation effect on insulin synthesis and secretion, given the presence of vitamin D receptors, 1α-hydroxylase, and vitamin D-binding protein in pancreatic islet cells [14, 33, 39]. Vitamin D has additional receptors in adipocytes, muscle, and hepatocytes reducing insulin resistance by enhancing insulin receptor expression and insulin responsiveness for glucose transport, and regulating calcium metabolism [14, 33, 37, 39, 40]. In this line, cholecalciferol (the active form of vitamin D) has the ability to increase calcium influx across β-cells’ membrane into their intracellular space, influencing insulin secretion [14, 33, 37–40].

The potential role of vitamin D supplementation in diabetes prevention has been also studied; however, inconclusive outcomes have been obtained [17, 19, 20, 33, 41]. The most important randomized trial (the D2d study) included 2,423 obese participants with prediabetes [17]. After 2.5 years of median follow-up, a new diagnosis of T2D was observed in 293 participants allocated to 4000 IU/d of vitamin D and in 323 participants of the placebo group, with hazard ratio of 0.88 (95% CI 0.75–1.04; *p* = 0.12). In subgroup analysis, participants showing insufficient levels of vitamin D seemed to have a lower risk of developing T2D after supplementation. The initial levels of vitamin D were 28 ng/ml and the trial’s duration probably was not very long [17]. Despite the lack of conventional statistical significance, this trial observed a 12% relative reduction in risk [17]. Given this finding, it can be speculated that a longer duration and a larger sample size would probably have found a greater benefit. In fact, this interpretation was favored by a secondary *per protocol* analysis, which found that among participants adherent to the trial protocol, vitamin D lowered the risk of developing T2D at the end of the study [42]. Another meta-analysis reported a 27% reduction in the risk of progression from prediabetes status to diabetes after supplementation in non-obese subjects (RR 0.73; 95% CI 0.57–0.92) [33]. In addition, Li X et al. evinced a partial reduction of insulin resistance compared with placebo in patients diagnosed with T2D [20]. However, this outcome was shown after large doses and short-term supplementation in vitamin-D-deficient non-obese participants from Middle East Asia [20]. In the same line, another randomized controlled clinical trial conducted among pre-diabetic Iranian individuals found a slightly but statistically significant improvement on insulin sensitivity and a lower risk of progression toward diabetes in the vitamin D group compared to placebo, after high dose supplementation [43]. Published literature highlights the significant association between serum vitamin D deficiency and diabetic peripheral and cardiac neuropathy, erectile dysfunction and diabetic retinopathy, as it has been suggested that vitamin D has a protective effect over the optic nerves [14, 37, 44, 45]. In addition, low serum 25(OH)D status has been related to an increased risk of developing diabetic nephropathy, after finding that vitamin D analogs strengthen the protective effects of the renin–angiotensin–aldosterone system inhibitors over the renal function through suppression of renin expression [37, 44].

In addition, other authors have studied the association between predicted serum vitamin D and other clinical outcomes, such as different types of tumors (breast or colorectal cancers) [46, 47] or the risk of fractures [48], among others.

Our study has some limitations. The use of self-reported, validated FFQ to evaluate nutritional intake provides subjective details and may fall into an information bias. This is particularly important when reporting vitamin D intake, since the data collected reflect a consumption below the recommended limits (15 mcg/day) [7]. In addition, our serum vitamin D predictive model is a subjective tool, which may be more useful in the epidemiological and research fields, rather than the daily clinical setting, as it may not be suitable for particular groups such as pregnant women, children or patients with severe kidney disease, in which specific prediction models should be used to reach adequate outcomes. In this line, considering that serum vitamin D concentrations show diurnal [49] and annual fluctuations (higher levels in summer and lower during winter), weakly but significantly correlate with other hormones such as testosterone and cortisol [50], assessing predicted serum levels of vitamin D at the same hour of the day and during the same season in a sufficiently large sample of participants with and without T2D, could strengthen the significance of our findings. Moreover, the sample is not representative of the general population as it includes mostly young adults with a high educational level. Therefore, the extrapolation of our results to general population should not be based on representativeness of the study sample. Proper generalization should be grounded on the knowledge of specific conditions and the clear understanding of involved biological mechanisms [51]. However, this feature of our design also has the advantage of increasing the internal validity of our results due to the high educational level and homogeneity of the participants, which reduces potential confounding related to educational and socioeconomic status. The strengths of the current study include its prospective design and dynamic participation. To our knowledge, this is one of the few studies to assess the association between estimated circulating vitamin D and incidental T2D in Spanish population and the first one based on a validated predictive model. The SUN project also includes a large sample with a considerable long-term follow-up and a good retention rate, which are relevant to ensure an adequate temporal sequence between exposure and outcome. Self-reported cases of incident T2D were confirmed by an expert endocrinologist who was blinded to the exposure, which provides data reliability and a high specificity. What is more, we adjusted the models for a wide range of potential confounders and several sensitivity analyses were performed to guarantee the robustness of our outcomes and minimize the likelihood of residual confounding.

## Conclusion

Our study based on validated predicted serum 25(OH)D in a Mediterranean cohort conclude that vitamin D has a role in the modulation of diabetes risk, suggesting that higher levels of predicted vitamin D at baseline may have a protective effect in the prevention of incident T2D. However, the baseline 25(OH)D threshold considered to be preventive from developing type 2 diabetes remains unclear.

### Supplementary Information

Below is the link to the electronic supplementary material.

Supplementary file 1 (DOCX 15 KB)

## Data Availability

The data from the SUN project that support our findings are available upon request to the Department of Preventive Medicine and Public Health, School of Medicine, University of Navarra (Spain) at sun@unav.es.

## Abbreviations

T2D: Type 2 diabetes
SUN project: Seguimiento Universidad de Navarra
BMI: Body mass index
FFQ: Food Frequency Questionnaire
MET: Metabolic equivalent of task
HR: Hazard ratio
CI: Confidence interval
d.f.: Degrees of freedom
IQR: Interquartile range
