# Supplementary material for: Vitamin D and risk of developing type 2 diabetes in the SUN project: a prospective cohort study
Source: J Endocrinol Invest. 2024 Mar 8;47(9):2313–23. doi: 10.1007/s40618-024-02324-3 (PMC11368983; doi:10.1007/s40618-024-02324-3)
Supplement: Supplementary file 1 — (DOCX 15 KB) [file 40618_2024_2324_MOESM1_ESM.docx]

**Supplementary material**

**Table 1.** Detailed stages included in the development of the multiple linear regression model.

| Steps | Variables | Linear regression model equation | Pearson correlation coefficient (95% CI) between linear prediction and serum vitamin D |
| --- | --- | --- | --- |
| 1 | Energy adjusted Vitamin D intake | Linear prediction 1 = 19.33 + (0.20 * vitd_a) ^a^ | 0.13 (0.001 to 0.26) |
| 2 | Age and sex | Linear prediction 2= 21.15 + (0.13 * vitd_a) +  (-0.06 * age) + (1.67 * sex) | 0.22 (0.09 to 0.34) |
| 3 | BMI | Linear prediction 3 = 29.57 + (0.11 * vitd_a) +  (-0.002 * age) + (2.06 * sex)+(-0.44 * bmi) ^b^ | 0.30 (0.17 to 0.41) |
| 4 | Skin reaction | Linear prediction 4 = 30.92 + (0.09 * vitd_a) +  (-0.02 * age) + (1.83 * sex) + (-0.43 * bmi) +  (-2.99 * skin2) ^c^ | 0.34 (0.22 to 0.45) |
| 5  6  7 | Walking  Summer sun exposure  Physical Activity | Linear prediction 5 = 27.90 + (0.06 * vitd_a) + (-0.04 * age) + (1.72 * sex) + (-0.36 * bmi) +  (-3.27 * skin2) + (0.04 * walk_min) ^d^  Linear prediction 6 = 26.16 + (0.06 * vitd_a) +  (-0.02 * age) + (1.29 * sex) + (-0.33 * bmi) +  (-3.61 * skin2) + (0.04 * walk_min) + (0.80 * sun1) ^e^  Linear prediction 7 = 24.4 + (0.04 * vitd_a) +  (-0.01 * age) + (1.37 * sex) + (-0.31 * bmi) +  (-3.71 * skin2) + (0.03 * walk_min)  + (0.77 * sun1) + (0.03 * phys_act) ^f^ | 0.37 (0.25 to 0.48)  0.41 (0.30 to 0.52)  0.43 (0.31 to 0.53) |

(a) vitd_a: Dietary vitamin D and supplementation, energy-adjusted by residual method (mcg/day), (b) bmi: body mass index (kg/m^2^), (c) skin2: skin reaction after sun exposure (0: mild;1: severe), (d) walk_min: walking time (minutes/day), (e) sun1: sun exposure during summer (hours/day), (f) phys_act: physical activity measured by metabolic equivalents (METs-h/week).
